# Authentic Leadership and Task Performance *via* Psychological Capital: The Moderated Mediation Role of Performance Pressure

**DOI:** 10.3389/fpsyg.2022.722214

**Published:** 2022-05-31

**Authors:** Eunmi Jang

**Affiliations:** College of Business, Honam University, Gwangju, South Korea

**Keywords:** authentic leadership, task performance, psychological capital, performance pressure, moderated mediation effect

## Abstract

Authentic leadership has received significant academic attention. It is now imperative to understand how authentic leadership’s effectiveness varies in different situations or conditions, which is vital to reestablishing it as an independent leadership theory. To this end, this study aims to verify the positive influence of authentic leadership on the task performance of members within an organization. Further, it seeks to confirm the situations that moderate the relationship between authentic leadership and task performance. Specifically, the mediating mechanism of psychological capital in this relationship, the moderating effect of performance pressure on the relationship between authentic leadership and psychological capital, and the moderated mediating effect are demonstrated. This study used a time-lagged survey to test the hypotheses; two online surveys were staggered by 1 month and completed by 485 participants in South Korea. The empirical analysis confirmed all the proposed hypotheses. First, authentic leadership was positively related to task performance. Second, psychological capital had a mediating effect on the relationship between authentic leadership and task performance. Third, task performance pressure was negatively related to the relationship between authentic leadership and psychological capital. Specifically, the strength of the indirect effect increased as the employee performance pressure decreased. Based on these results, various theoretical and practical implications are suggested for the extended application of the authentic leadership theory in organizations and future research directions are proposed.

## Introduction

Consistent performance outcomes are essential for a company to remain sustainable. Fierce competition is unavoidable in today’s rapidly changing business environment. Organizations demand higher levels of performance from their members to overcome market uncertainties caused by high competition (Loehr and Schwartz, 2001). As a result, members feel obligated to work for the sake of improving performance (Sitkin et al., 2011). However, are these performances sustainable in the long run? Many organizational theorists argue that short-term performance must be balanced with sustainable long-term objectives for an organization to be sustainable (e.g., Levinthal and March, 1993; Sutcliffe and Sitkin, 2000). Many problems may arise when leaders merely chase after short-term profits and fail to manage the organization from a long-term profit perspective.

Similarly, many problems emerge from self-interested leaders in organizations seeking personal profits. The need for leadership that contributes to organizational performance from a sustainable and long-term perspective has given rise to authentic leadership (Caza and Jankson, 2011). Authentic leadership is rapidly emerging as a new type of leadership that can address the challenges currently faced by many organizations (George, 2003; Luthans and Avolio, 2003; Gardner et al., 2005; Walumbwa et al., 2008).

Until date, several studies have been conducted in the context of developing authentic leadership theory as an independent leadership theory, and the pursuit is still actively underway. The research trend concerning authentic leadership can be classified into three main directions. First, there are studies that have identified authentic leadership’s sub-dimensions and suggest ways to develop them further (Cooper et al., 2005). Second, research has been conducted on the relationships among authentic leadership, organizational performance, and outcome variables, including several theoretical (George, 2003; Avolio et al., 2004; Gardner et al., 2005; Ilies et al., 2005) and empirical studies (e.g., Walumbwa et al., 2010; Laschinger et al., 2015; Leroy et al., 2015). The research results have demonstrated that authentic leadership has a positive influence on members’ performance and organizational effectiveness (Price, 2003; Walumbwa et al., 2007; Yammarino et al., 2008; Zhu et al., 2011). Third, studies have been conducted on authentic leadership’s dynamics to examine situations and processes in which authentic leadership can affect various outcome variables, including performance (e.g., Walumbwa et al., 2010; Peus et al., 2012; Wang et al., 2014; Gardner et al., 2021).

This study aims to achieve the following research objectives. First, this study seeks to reconfirm authentic leadership’s positive influence on employee’s task performance. Although there are still very few studies on the effect of authentic leadership on performance, previous studies have revealed that subordinates respond to leader authenticity by generating better performance as an individual (e.g., Wang et al., 2014; Ribeiro et al., 2018a). Therefore, it is crucial for business management research to understand authentic leadership’s influence on performance.

Second, this study seeks to verify psychological capital’s mediating effect on the relationship between authentic leadership and task performance. Various mechanisms have been proposed to explain the process by which authentic leadership influences performance. Examples include empowerment (Walumbwa et al., 2010), trust (Clapp-Smith et al., 2009), job engagement (Giallonardo et al., 2010), and social identity (Wong et al., 2010). However, authentic leadership contributes to the formation and development of members’ psychological capital, given the importance of the positive interactions between authentic leaders and members during the growth process (Luthans and Youssef, 2004). Authentic leadership involves a high level of psychological capital (Petersen and Youssef-Morgan, 2018). Furthermore, it increases members’ psychological capital (Avolio et al., 2004; Gardner and Schermerhorn, 2004; Ilies et al., 2005). Thus, psychological capital works as an important mechanism in the process by which authentic leadership affects performance (Gardner and Schermerhorn, 2004). Moreover, only a few studies have focused on psychological capital’s mediating effect on the relationship between authentic leadership and performance (Woolley et al., 2011).

Third, this study attempts to demonstrate the moderating effect of “performance pressure” on the relationship between authentic leadership and psychological capital. The concept of authentic leadership, which transpired from practical interest, has shown significant progress theoretically. However, its effectiveness is still being questioned compared to other leadership theories (Cooper et al., 2005; Yammarino et al., 2008). It has been suggested that research must be conducted in various organizational situations for authentic leadership to be established as a more sophisticated leadership theory (Gardner et al., 2011). Therefore, situational variables must be considered. Petersen and Youssef-Morgan (2018) explored authentic leadership’s antecedents and highlighted the need to include the influence of organizations’ situational variables on authentic leadership in future studies. Situational factors’ active consideration plays an important role in the development of authentic leadership research (Caza and Jankson, 2011).

Particularly, “situations where authenticity is sacrificed” (Guenter et al., 2017) may occur with authentic leadership in which authenticity is the key. As such, an empirical study observing the situational factors associated with the factors that hinder authentic leadership’s effectiveness in an organization is urgently required. This is in consideration of the situations where authentic leadership’s effectiveness continues to be questioned. The authentic leader’s positive influence may be unrecognized by members or weakened due to certain situational variables; in such cases, their influence loses effectiveness (Duffy et al., 2002).

In this context, the performance pressure prevalent in domestic companies is seen as a situational factor that can undermine the effectiveness of leadership. This study seeks to confirm how it moderates the relationship between authentic leadership and the development of members’ psychological capital. Many companies are under pressure to produce maximum performance in a short period of time amid the rapidly changing business environment. Performance pressure can serve as a driver for leaders to improve their members’ performance. However, it can also be a huge burden, which consequently negatively affects members’ motivation and development. In climates where companies demand financial performance in the market economy, leaders are compelled to focus only on managerial styles, not on authenticity. As such, it hinders the creation of a sustainable competitive advantage (Yoon, 2012). This can result in situations that reduce authentic leadership’s effectiveness, which otherwise improves companies’ long-term sustainability.

In summary, this study aims to verify psychological capital’s mediating effect on how authentic leadership affects task performance. Moreover, it looks into performance pressure’s moderating effect on the relationship between authentic leadership and psychological capital. This will contribute to authentic leadership’s conceptual development by validating the mediating and moderating effects that have rarely been studied in the research on authentic leadership. Furthermore, the present study provides practical implications for authentic leadership’s development and application.

## Theory and Hypotheses

### Authentic Leadership

Authentic leadership can be considered a root construct for the development of positive leadership involving ethical, servant, and transformational behaviors (Avolio and Gardner, 2005; Walumbwa et al., 2008). This implies that authentic leadership is a broader leadership concept that encompasses many positive leadership styles. In other words, as leadership’s foundation, authenticity is authentic leadership’s core value. Authenticity means having a clear and firm knowledge of oneself in every aspect (e.g., beliefs, preferences, strengths, and weaknesses) and acting consistently through self-awareness (Gardner et al., 2005; Ilies et al., 2005). Thus, authentic leadership is a psychological competence that encourages positive moods and abilities based on authenticity. Moreover, it can be defined as the behavior pattern of leaders who develop themselves and their members based on four subfactors: self-awareness, relational transparency, balanced information processing, and internalized moral perspective (Walumbwa et al., 2008).

Early studies on authentic leadership describe authentic leaders as those who value the respect for themselves over their personal roles as leaders (Hoy and Henderson, 1983). They want to understand who they are and show consistent behaviors based on the values and beliefs created in this process. As such, they do not act intentionally as leaders (George, 2003; Avolio et al., 2004; Gardner et al., 2005). Authenticity is a behavior that is revealed through repeated acts of self-awareness and reflection. An authentic leader acts with authenticity as a core value. This behavior is important because a leader knows themselves better than anyone else. Such a leader has a high level of self-esteem that is not easily influenced by any situation (Kernis, 2003).

A high level of optimal self-esteem allows the leader to freely share the information needed for decision-making with the organization’s members. This will also enable the leader to willingly accept members’ opinions, thereby promoting open communication. The leader’s behaviors, abilities, and internalized morality that are manifested based on values and beliefs are recognized by members. This helps them make an accurate judgment about their leader (Walumbwa et al., 2010). An authentic leader makes self-expression consistent with their self-concept (Shamir and Eilam, 2005). Such a leader’s subordinates imitate the expressions and behaviors formed through self-awareness and self-regulation (Luthans and Avolio, 2003). The interaction between the members and an authentic leader, based on the leader’s behavior, encourages members to view their leaders as role models and leads to authentic leadership. These behaviors are very effective in eliciting positive organizational performance and desirable behaviors among the members (Avolio and Gardner, 2005). Most importantly, this effectiveness can be maintained if the leader’s behavior is consistent.

Walumbwa et al. (2008) proposed authentic leadership’s four sub-factors: self-awareness, balanced information processing, relational transparency, and internalized moral perspective. These widely accepted constructs constitute the instrument, “Authentic Leadership Questionnaire” (ALQ).

First, self-awareness means that leaders know themselves very well (Campbell et al., 1996) and understand how behaviors exercised through self-awareness affect members. Therefore, self-awareness influences leaders’ thinking, inspirational motivation, and behaviors. Moreover, authentic leaders with a high level of self-awareness are more capable of leading members and helping them grow.

Second, balanced information processing refers to objective thinking through the positive and negative aspects of problem-solving. Open methods are used for decision-making, and highly relevant information is utilized to facilitate objective decision-making. For example, leaders closely analyze relevant facts and tend to involve members in the decision-making process by seeking and listening to their various opinions before undertaking important decisions. Such authentic leaders’ characteristics give members the perception that leaders assert their own opinions and, at the same time, actively listen to other members’ opinions.

Third, relational transparency involves acting on one’s true nature (not a manipulated or false one) to honestly share a set of information such as a leader’s true thoughts and emotions. Leadership behavior that promotes positive relationships is demonstrated by drawing the corresponding approvals or opposing opinions from members. Consequently, this behavior by an authentic leader increases openness, responsibility, and integrity among the leader and the members. It also further enhances the social exchange relationship between them as the members’ as well as the leader’s expectations become clearer.

Fourth, an internalized moral perspective means that true leaders regulate themselves from a moral perspective and act according to their respective norms (Lemoine et al., 2019). This means that they exhibit a high level of moral behavior based on internalized moral standards and values and not external social pressures. Therefore, authentic leaders encourage themselves, the members, and their organizations to think from an ethically broader and deeper perspective when faced with serious ethical issues (Werhane and Freeman, 1999).

### Authentic Leadership and Task Performance

Task performance is the level of achievement in official tasks assigned to members (Stumpf et al., 1983). It is an act of performing the role presented in the job description (Williams and Anderson, 1991). Moreover, it involves the concept of in-role behavior that must be performed by members. Members’ task completion is directly related to the organization’s effectiveness and personal job achievement (Son et al., 2013). Therefore, as researchers have continued to study the antecedents of task performance, leadership has drawn attention for a long time as an antecedent of members’ task performance (Hwang et al., 2015).

Authentic leadership can have a positive influence on members’ task performance as members perceive authentic leaders as attractive role models. They have the expectation and conviction that their leaders do the right thing (Shamir and Eilam, 2005). Thus, they continue to imitate their leaders, who are seen as exemplary and ideal role models (Avolio et al., 2004). Walumbwa et al. (2010) argued that through exemplary behaviors, authentic leaders can be attractive and trustworthy role models for their members. Authentic leaders try to help members maintain their views on what they think is right (Walumbwa et al., 2008). Considering this approach, leaders support members in making their own decisions through open dialog, rather than through unilateral task instruction (Ilies et al., 2005). Further, leaders provide a transparent decision-making process instead of forcing their own opinions on members or just accepting other members’ biased opinions (Avolio and Gardner, 2005). Independent decision-making by members and leaders is important in performing individual tasks. Argote (1999) stated that members made good use of resources when they had easy access to diverse information. In such circumstances, members were more likely to complete their assigned tasks. Kernis (2003) and Kernis and Goldman (2005) found that those who perceived themselves as having a higher level of authenticity, demonstrated a higher level of enthusiasm in the pursuit of goals and decision-making. Authentic leaders’ role modeling will also encourage members to be more immersed and engaged in tasks, thereby improving their task performance.

In summary, the authentic leader’s behavior, based on authentic leadership values, is consistent (Kernis, 2003; Gardner et al., 2005). Thus, members consider them as their model and pursue task performance. Particularly, members’ behavior is also consistent since authentic leadership’s influence does not change easily and lasts for a long time (Avolio and Gardner, 2005). This leads to sustainable performance. Extant literature on the effect on performance is relatively scarce (Avolio et al., 2004), but recent studies from around the world have proven that authentic leadership is positively related to employee performance (e.g., Clapp-Smith et al., 2009; Wang et al., 2014; Leroy et al., 2015; Ribeiro et al., 2018a b; Duarte et al., 2021). Based on the logic and results of these prior studies, the following hypotheses were established:

*Hypothesis* 1: The extent of authentic leadership is positively related to followers’ perceived task performance.

### The Mediating Role of Perceived Psychological Capital in the Relationship Between Authentic Leadership and Task Performance

The conceptual development of psychological capital has been underway since human resource management researchers began focusing on members’ positive and healthy internal energy, given the growth of positive psychology (Luthans et al., 2006). Psychological capital is not a single concept. Rather, it consists of four sub-factors—hope, self-efficacy, resilience, and optimism—which play a very important role as a source of people’s inner motivation by converging with each other and not acting as individual effects (Luthans et al., 2007; Avey et al., 2010).

Authentic leadership is a concept that fundamentally encompasses psychological capital (Cooper et al., 2005). Authentic leaders are in a positive psychological state accompanied by optimal self-esteem and psychological well-being. Additionally, an authentic leader’s positive psychological state is shifted to members through role modeling, further facilitating the development of such states (Gardner et al., 2005). According to the social learning theory of Bandura (1977), an authentic leader can spread their internalized values to members during an interaction. Meanwhile, members can internalize the leader’s values with increased psychological capital through positive role modeling (Gardner et al., 2005). Authentic leaders are perceived as role models through consistency, fairness, and transparency. Thus, members see what behaviors are effective and desirable (Bandura, 1977; Salancik and Pfeffer, 1978). Further, authentic leaders’ values are transferred and learned by members. Therefore, authentic leaders play a key role in forming psychological capital for their members (Avolio et al., 2004). Similarly, authentic leadership has a positive influence on members’ psychological capital.

Authentic leadership can also have a positive influence on each of psychological capital’s four sub-factors. Authentic leaders try to answer members’ questions and provide feedback with an open attitude. This process helps members realize their abilities (Khan, 2010) and increases their self-efficacy. Members with an increased self-efficacy will be able to accept challenging tasks. These members perform well under pressure and stress. Authentic leaders motivate members to set goals, decide strategies for their goals, and ultimately achieve them (Khan, 2010). Therefore, authentic leadership has a positive influence on hope, which can be considered a combination of the agency to suggest goals and pathways to attain them (Snyder et al., 2002). Furthermore, authentic leaders attempt to be more proactive and respond appropriately to given situations as role models when members are faced with difficult problems (Luthans and Avolio, 2003). These positive problem-solving approaches are internalized by members, improving their resilience to positively approach the problem’s source or prospects. Resilience will also allow them to quickly recover from problems and is linked to positive expectations for the future. Moreover, positive expectations lead to significant differences in individual performances (Camfield and McGregor, 2005). Authentic leaders maintain a positive psychological state based on their firm values and beliefs about themselves. They also endure difficulties or frustrations or recover from them quickly. These leaders’ images are propagated and learned by members through observation and inspiration.

Recently, an increasing number of studies have examined psychological capital’s mediating effect on authentic leadership’s influence on member attitudes and behaviors (Calheiros, 2012; Hu et al., 2018; Armstrong and Cassidy, 2019; Ciftci and Erkanli, 2020). For example, psychological capital as a mediator, Armstrong and Cassidy (2019) showed that authentic leadership has a negative effect on job stress. Ciftci and Erkanli (2020) demonstrated psychological capital’s mediating effect between authentic leadership and job enthusiasm, while Hu et al. (2018) demonstrated psychological capital’s mediating effect on authentic leadership’s influence on proactive behavior. Studies have also been conducted at the group level. Calheiros (2012) focused on authentic leadership and disruptive leadership, arguing that psychological capital at the group level affected team performance, suggesting the need for empirical research. Walumbwa et al. (2010) showed the group-level psychological capital’s mediating effect on the process by which authentic leadership influences group performance and collective citizenship behavior. These studies commonly maintain that authentic leadership influences a member’s attitude and behavior, mediated by the member’s psychological capital.

According to the broaden-build theory, positive experiences expand members’ thinking and behavior while encouraging them to take on challenges and adventures. Therefore, similar to positive energy, a high level of psychological capital drives members to produce high performance (Fredrickson, 2001; Hobfoll, 2002; Gooty et al., 2009). Psychological capital is also an important resource for positive organizational behavior (Avey et al., 2010). It plays a key role in explaining members’ performance (Luthans et al., 2006; Norman et al., 2010; Peterson et al., 2011). Members with greater psychological capital try harder with the conviction that they can achieve better performance (self-efficacy), derive multiple ways to solve problems with a strong willpower (hope), expect positive results based on internal attribution (optimism), and make efforts to respond positively to difficult situations (resilience). Thus, psychological capital provides inspirational motivation to achieve goals and perform better (Avey et al., 2011). Many prior studies have demonstrated psychological capital’s positive influence on members’ performance (Avey et al., 2010; Walumbwa et al., 2010; Peterson et al., 2011).

In summary, authentic leaders develop members’ psychological capital, including self-efficacy, hope, optimism, and resilience. This is done through the process of role modeling based on authenticity and by inducing intrinsic motivation, such that members behave desirably for the organization. This intrinsic motivation improves members’ task performance. Based on this logic and the results of prior research, the following hypothesis is proposed regarding psychological capital’s mediating effect:

*Hypothesis* 2: The relationship between the extent of authentic leadership and followers’ task performance is mediated by followers’ perceived psychological capital.

### The Moderating Role of Performance Pressure in the Relationship Between Authentic Leadership and Psychological Capital

Authentic leadership’s meaning and effectiveness can vary according to context (Chan, 2005). Scheepers and Elstob (2016) recommended exploring contextual variables to confirm authentic leadership’s effectiveness. Hence, this study aims to examine what causes a moderating effect on the relationship between authentic leadership and psychological capital. This is achieved by selecting performance pressure as a contextual variable, which is emphasized by many companies for survival.

Performance pressure collectively refers to the negative attitude of assessing that one’s performance will be unsatisfactory. It is the belief that the current performance will be insufficient to achieve goals and also considers the associated negative emotions (Zimbardo and Leippe, 1991). Performance pressure also affects an individual’s beliefs in the negative outcomes of failing to achieve desired goals (Zimbardo and Leippe, 1991; Eisenberger and Aselage, 2009). This raises concerns regarding promotions, pay increases, and other work benefits that will be lost if performance is not achieved (Mitchell et al., 2018). The psychological burden from the perceived judgment of the target level of achievement will be greater if a member recognizes that the time available to perform a task is too short. Consequently, this may promote maladaptive behaviors, such as seeking easier tasks or giving up, or may negatively affect individuals’ psychological well-being and health (Ames, 1992; Roberts and Nerstad, 2020).

Due to their consistent behavior and fair attitudes, authentic leaders serve as role models, which draws effective and desirable behaviors from members (Bandura, 1977; Salancik and Pfeffer, 1978). Members are motivated to choose an authentic leader as their role model and act in the same manner as the leader. Such behaviors play a key role in developing members’ psychological capital (Avolio et al., 2004). However, when high performance pressure is perceived, the motivation to follow an authentic leader’s behavior may be overtaken by the motivation to avoid external risks. This is because poor performance is believed to be primarily punished in an organization (e.g., Newton and Duda, 1999; Raub and Robert, 2013). Even when incentives are provided for high performance, performance pressure motivates members to participate in extrinsic compensation more than the motivation to follow an authentic leader’s behavior. As such, reliance on extrinsic control is encouraged. However, performance pressure in general is perceived as a burden of punishment for poor performance rather than as a reward expectation for high performance (Zimbardo and Leippe, 1991). Further, the motivation to avoid performance pressure is likely to be stronger.

The greater the performance pressure, the more likely a member will find it difficult to control the situation or meet the job demands, ultimately producing negative outcomes and experiencing greater internal and psychological pressure (Mitchell et al., 2018). In these situations, members tend to rely on the organization’s direct control to avoid any external threats before choosing their own authentic leader who is not pretentious and ensuring the leader’s optimal self-esteem and psychological well-being. Therefore, the authentic leader’s influence is relatively small. Low performance pressure encourages authentic leadership’s active social learning with a significant influence on members’ psychological capital. However, high performance pressure hinders authentic leadership’s social learning through extrinsic control, reducing the influence on members’ psychological capital. Based on this logic, the following hypothesis is proposed regarding performance pressure’s moderating effect on the relationship between authentic leadership and psychological capital:

*Hypothesis* 3: Performance pressure negatively moderates the relationship between authentic leadership and psychological capital. The higher the performance pressure, the weaker the positive relationship between authentic leadership and psychological capital.

**Figure 1:**
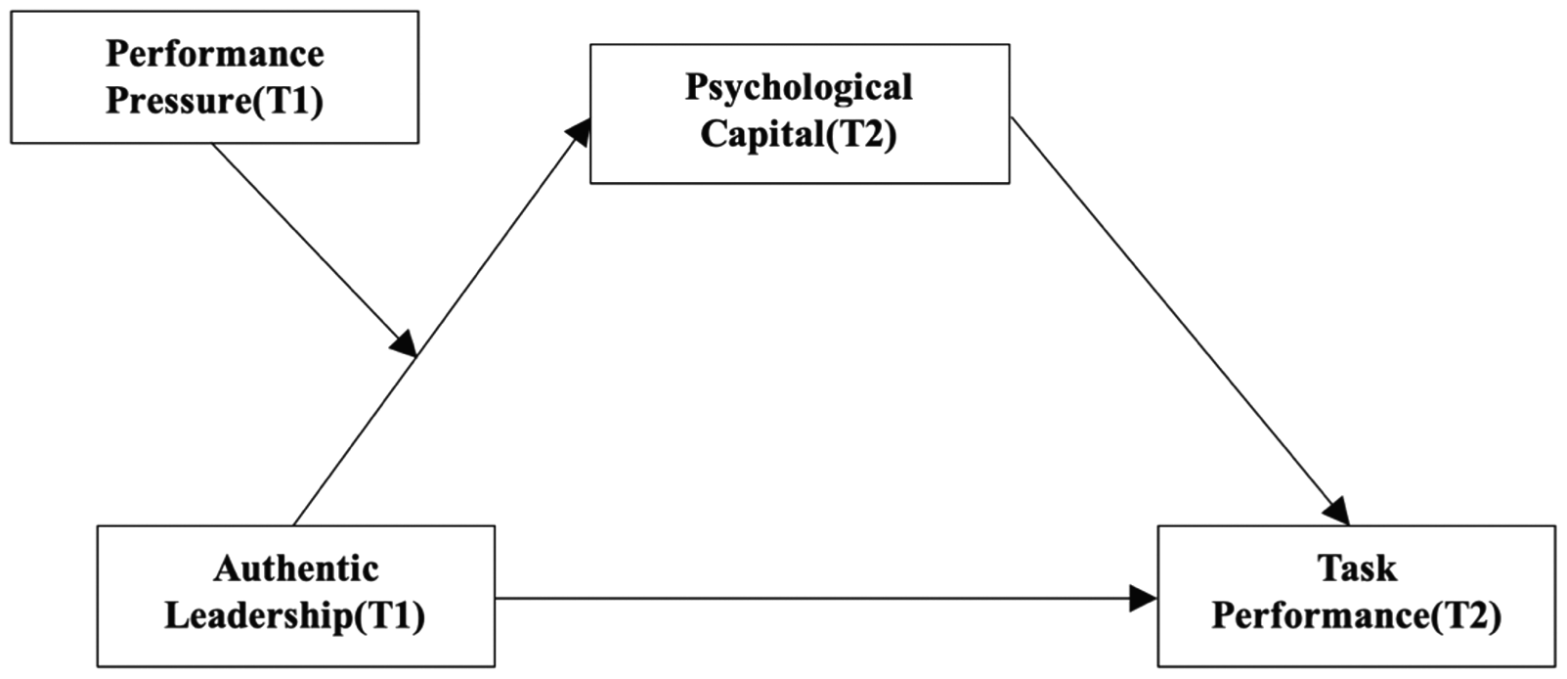
Theoretical model of this study.

Assuming that performance pressure, as perceived by members, moderates the relationship between authentic leadership and psychological capital, it can be inferred that performance pressure can conditionally affect psychological capital’s mediating effect on the relationship between authentic leadership and task performance. In other words, the mediating effect between the study variables, as presented in the theoretical model in Figure 1, is verified. The higher the perceived performance pressure, the greater the effect on the mediating role of psychological capital and the weaker the positive influence of authentic leadership on task performance. Therefore, the following hypothesis is established:

*Hypothesis* 4: The indirect association between authentic leadership and followers’ task performance through psychological capital is conditionally dependent on the levels of perceived performance pressure, such that the indirect association is weakened under a high level of performance pressure.

The research model based on the above hypotheses is depicted in Figure 1.

## Materials and Methods

### Sample and Data Collection

The participants were full-time employees working for private companies in South Korea. The survey was conducted through Macromill Embrain, a credible online data collection platform with 6.4 million survey panels in Korea. Data were collected in this study over two stages (Time 1 and Time 2) to prevent common method bias (CMB).

Perceptions of authentic leadership and performance pressure were measured at Time 1. Psychological capital and task performance were then measured a month later for members who responded at Time 1. The method of hiring a specialized survey institute, Macromill Embrain, was applied because surveys for longitudinal studies require precision in collecting data from the same members at the same time interval.

At Time 1, a total of 608 people responded; a month later, 489 people responded in the second stage (i.e., at Time 2). Based on the data of 489 respondents who responded to both the surveys conducted at Time 1 and Time 2, the data obtained from 485 respondents (excluding four missing values) were used in the final analysis. The respondents’ demographic characteristics are as follows. There were 247 men (50.9%) and 238 women (49.1%). Concerning age, 92 people were in their 20s (18.9%), 215 in their 30s (44.3%), 135 in their 40s (27.9%), and 43 people were in their 50s or older (8.9%), with the highest distribution of people in their 30s. Regarding educational background, there were 23 high school graduates (4.7%), 76 college graduates (15.7%), 317 university graduates (65.4%), 61 master’s degree holders (12.6%), and 8 doctorate degree holders (1.7%). In terms of position, there were 254 staff members or assistant managers (27.8%), 109 managers (22.5%), 54 deputy general managers and general managers (22.5%), and 22 executives (4.5%). The number of years of service was 1–4 years for 249 people (51.4%), 5–9 years for 109 people (26.4%), 10–14 years for 54 people (13.5%), and 15 years or more for 22 people (8.7%).

### Measures

The questionnaire used a five-point Likert scale with response options ranging from 1 (strongly disagree) to 5 (strongly agree). The questionnaires, originally developed in English, were translated into Korean. We used a standard translation and back-translation procedure (Brislin, 1980) to ensure the research tool’s reliability and validity.

#### Authentic Leadership

Sixteen questions from the Authentic Leadership Questionnaire (ALQ; Walumbwa et al., 2008) were used to survey authentic leadership at Time 1. Specifically, the questionnaire included “My leader encourages everyone to speak their mind,” “My leader demonstrates beliefs that are consistent with actions,” and “My leader analyzed relevant data before coming to a decision,” among others. The Cronbach’s alpha value of the survey response was 0.96. As such, it meets the reliability criterion of 0.70 (Nunnally and Bernstein, 1994).

#### Performance Pressure

The four questions suggested by Mitchell et al. (2018) were modified and translated to survey performance pressure at Time 1. Specifically, the survey questions included “The performance pressure in my workplace is high,” “I feel tremendous pressure to produce results,” “If I do not produce at high levels, my job will be at risk,” and “I would characterize my workplace as a results-driven environment.” The Cronbach’s alpha value of the survey response was found to be 0.90.

#### Psychological Capital

Psychological capital was surveyed at Time 2 using 12 questions from the PsyCap Questionnaire Self-Rater Short Form (four questions on efficacy, four questions on hope, three questions on resilience, and two questions on optimism). Specifically, the survey questions included “I feel confident in representing my work area in meetings held with the management,” “I can think of many ways to reach my current work goals,” and “I usually take stressful things at work in stride,” among others. The Cronbach’s alpha value of the survey response was found to be 0.93.

#### Task-Performance

Task performance was surveyed at Time 2 using the four questions on in-role behavior developed by Williams and Anderson (1991). Specifically, the survey questions included “adequately completes assigned duties,” “fulfills the responsibilities specified in the job description,” and “performs tasks that are expected of them.”

#### Control Variables

Based on prior studies, the demographic characteristics that were assumed to affect the measurement variables, including age, position, years of service, educational background, and gender, were considered as control variables and surveyed at Time 2. Age and position are believed to affect members’ behavior with regard to task progress as their position advances over time. Furthermore, the knowledge and experiences relevant to a task accumulate over time (Wu and Parker, 2017). Education was considered to have an effect on employees’ behavior due to differences in knowledge level. Moreover, gender was added as a control variable since Woolley et al. (2011) confirmed it to be a situational variable between authentic leadership and positive organizational climate.

### Analysis Strategy

Stata 16.1 (Stata Corp., College Station, TX, United States) statistical software was used to conduct all the analyses in this study. First, prior to hypothesis testing, confirmatory factor analysis was performed to verify the validity of the constructs of the main variables of this study (Table 1). Thereafter, the variables were created by calculating the average value of the factors for which validity was secured. The mean and standard deviation are presented to check whether the variables used in the research model follow a normal distribution. In addition, the results of the correlation analysis of the variables and Cronbach’s alpha values for each variable are presented (Table 2). As a result, most of the correlations between variables coincided with the direction predicted by the hypotheses.

**Table 1:** Chi-square difference tests and goodness-of-fit statistics for alternative measurement models.

Measurement Model	*χ* ^2^	*df*	RMSEA	CFI	TLI	SRMR	Δ*df*	Δ*χ*^2^
4-Factor model	1866.93^***^	588	0.07	0.90	0.89	0.06	-	-
3-Factor model	4255.41^***^	591	0.11	0.71	0.69	0.14	2388.48	3.00
2-Factor model	5486.52^***^	593	0.13	0.61	0.59	0.15	1231.11	2.00
1-Factor model	6637.19^***^	594	0.15	0.52	0.49	0.16	1150.67	1.00

4-Factor model (hypothesized model), 3-Factor model (authentic leadership and psychological capital merged), 2-Factor model (authentic leadership, psychological capital, task performance merged), and 1-Factor model (all variables merged). RMSEA, Root Mean Square Error of Approximation, CFI, Comparative Fit Index, TLI, Tucker-Lewis Index, SRMR, Standardized Root Mean Square Residual; and Source: Stata software analysis.

*** *p* < 0.001.

**Table 2:** Means, standard deviations, correlations, and consistency coefficients of the variables.

Variables	Mean	SD	1	2	3	4	5	6	7	8	9
1. Gender	0.49	0.50	-								
2. Age	37.46	8.37	−0.35^***^	-							
3. Education	2.91	0.73	−0.15^***^	0.08	-						
4. Job level	2.64	1.46	−0.41^***^	0.66^***^	0.17^***^	-					
5. Tenure	2.73	1.17	−0.21^***^	0.45^***^	0.05	0.41^***^	-				
6. Authentic leadership	3.28	0.78	0.01	0.09	0.09	0.10	0.05	(0.96)			
7. Psychological capital	3.47	0.65	−0.12^**^	0.33^***^	0.12^**^	0.36^***^	0.20^***^	0.43^***^	(0.93)		
8. Task performance	3.79	0.69	0.15^***^	0.07	0.08	0.11	0.07	0.22^***^	0.46^***^	(0.91)	
9. Performance pressure	3.06	0.89	−0.14^**^	0.12^**^	0.05	0.21^***^	0.13^**^	0.07	0.16^***^	0.06	(0.90)

Gender (0 = male, 1 = female), () = Cronbach’s alpha coefficients.

** *p* < 0.01;

*** *p* < 0.001.

The following methods were implemented to verify the hypotheses. First, a least-squares hierarchical multiple regression analysis was performed to verify Hypotheses 1–3. For Hypothesis 2, which is concerned with the mediating effect, the low statistical power issue observed in Baron and Kenny (1986) causal steps approach was found. Thus, the following method was suggested to provide the best balance between type 1 error and statistical power. In the first step, the independent mediator must be statistically significant (*α*|0). In the second step, the mediator must be statistically significant for the dependent variable, and the independent variable’s effect must be moderated (*β*|0). For the accurate verification of the mediating effect, the significance of the indirect effect was directly verified by confirming the indirect effect in the bootstrapping method. The bootstrapping method is accepted as a better method than the conventional Sobel test to verify the mediating effect. This is because no normal distribution is assumed, and type I errors are not presented. Bias-corrected two-tailed test results were obtained through the sampling process that was repeated 10,000 times. These were considered to be more accurate than the generalized results.

Lastly, the bootstrapping technique recommended by Preacher et al. (2007) was adopted to test Hypothesis 4. In this study, point estimates, standard errors, z-statistics, and 95% confidence intervals (percentiles and bias-corrected percentiles) were provided by iterating the bootstrap 10,000 times.

### CMB and Validity Check

The potential problem of CMB was reduced in the analysis by using the data collected with a one-month lag. However, it was still possible that CMB existed in the results’ analysis, since the data for all variables were obtained from the same source (i.e., members). Harman’s single-factor test was conducted to confirm the possibility of CMB. In general, when CMB is serious, one variable accounts for a large portion of the variation in the second variable. The first factor that represented the largest eigenvalue only accounted for 28% of the overall distribution when Harman’s single factor test result was examined. This indicates a low degree of CMB.

Before testing the hypotheses, confirmatory factor analysis was performed on the four factors: authentic leadership, psychological capital, task performance, and performance pressure. This was done to confirm the validity of the variables suggested in this study.

A model can be considered adequate when the comparative fit index (CFI) and Tucker-Lewis index (TLI) are 0.90 or higher, the root mean square error of approximation (RMSEA) is 0.08 or lower, and the ratio between *χ*^2^ (CMIN) and the degree of freedom is 3 or lower (Hu and Bentler, 1999; Kline, 2005). As shown in Table 1, the fit of the model presented in this study (four-factor model) is 1866.93, *df* = 588, *p* < 0.01, RMSEA = 0.07, CFI = 0.90, TLI = 0.89, and SRMR = 0.006. This indicates that all the fit indices are acceptable. The fit indices of the four-factor model are generally found to be inadequate in the one-factor model. Therefore, the four-factor model suggested in this study can be considered more reasonable than other factor models (Hu and Bentler, 1999).

## Results

### Descriptive Statistics, Correlations, and Reliability

Prior to testing the hypotheses, elementary statistics and correlation analyses were conducted in addition to the reliability analysis. Cronbach’s *α* was measured to verify reliability. The results indicate that all the variables observed in the model are 0.8 or higher, as shown in Table 2. The variables’ means, standard deviations, and correlations were analyzed. Correlations between the variables are mostly consistent with those proposed in the hypotheses. Specifically, there is a positive correlation between authentic leadership and task performance (*r* = 0.22, *p* < 0.001). Psychological capital is found to have a positive correlation with authentic leadership (*r* = 0.43, *p* < 0.001) and task performance (*r* = 0.46, *p* < 0.001). This is in agreement with prior studies’ results showing that members who highly perceive authentic leadership have a high level of psychological capital, contributing to task performance. Performance pressure is positively correlated with psychological capital (*r* = 0.16, *p* < 0.001). However, it exhibits no significance for authentic leadership (*r* = 0.07, n. s.) and task performance (*r* = 0.06, n. s.).

### Test of Hypotheses

Hierarchical multiple regression analysis was conducted to test the hypotheses presented in this study’s model. The results are presented in Table 3. First, the regression analysis results of authentic leadership’s influence on task performance are explained in this section. The regression coefficient of authentic leadership is significant in the positive direction (β = 0.19, *p* < 0.001) after controlling for the demographic variables in Model 1 to confirm the prediction of Hypothesis 1 and adding authentic leadership to Model 2. Additionally, the explanatory power of Model 2 increases significantly compared to Model 1 (Δ*R*^2^ = 0.04; *F* = 9.35, *p* < 0.001). Based on these results, Hypothesis 1 is supported.

**Table 3:** Results of the hierarchical multiple regression analysis of the study variable’s effects on task performance and psychological capital; standardized coefficients (*n* = 485).

Variables	Dependent variables					
	Task performance			Psychological capital		
	Model 1	Model 2	Model 3	Model 4	Model 5	Model 6
*Control variables*						
Gender	0.25^***^	0.24^***^	0.23^***^	0.03	0.03	0.04
Age	0.02	0.01	−0.05	0.142^**^	0.15^**^	0.15
Education	0.09	0.07	0.05	0.04	0.04	0.04
Job level	0.16^**^	0.15^*^	0.05	0.22^***^	0.21^***^	0.21^***^
Tenure	0.04	0.04	0.03	0.03	0.02	0.03
*Independent variable*						
Authentic leadership		0.19^***^	0.01	0.39^***^	0.38^***^	0.39^***^
Performance pressure					0.07	0.07
*Interactions*						
Authentic leadership × Performance pressure						−0.08*
*Mediator*						
Psychological capital			0.48^***^			
*F*	7.04^***^	9.35^***^	24.65^***^	34.43^***^	30.11^***^	27.03^***^
*R* ^2^	0.06	0.10	0.27	0.30	0.31	0.32
*R*^2^ change	-	0.04	0.17	-	0.04	0.01
VIF	1.50	1.42	1.47	1.42	1.38	1.33

Two-tailed tests of significance.

* *p* < 0.05;

** *p* < 0.01;

*** *p* < 0.001.

Second, the test result of Hypothesis 2 on the mediating effect of psychological capital on authentic leadership and task performance is explained here. Model 1 (Table 3) only observes the control variables’ influence on task performance, while Model 3 includes the surveyed authentic leadership value and perceived psychological capital. The explanatory power of Model 3 is much greater than that of Models 1 and 2 (∆*R*^2^ = 0.27, *F* = 24.65, *p* < 0.001). The mediating effect is tested in accordance with MacKinnon et al. (2007). This study confirms a statistically significant correlation between the independent variable (authentic leadership) and the mediator (psychological capital; see Model 4; *β* = 0.39, *p* < 0.001). Additionally, a significant correlation is confirmed between the mediator (psychological capital) and the dependent variable (task performance; *β* = 0.48, *p* < 0.001) under the independent variable’s influence (authentic leadership control). The method of verifying the mediating effect in accordance with MacKinnon et al. (2007) is validated because the results of the two steps are statistically significant.

Additionally, the mediating effect is validated using bootstrapping to overcome the limitation of quantifying the indirect effect (Preacher and Hayes, 2004). As seen in Table 4, the upper and lower bounds of the 95% confidence intervals (bias-corrected and accelerated) for psychological capital’s mediating effect between authentic leadership and task performance are 0.14 and 0.25, respectively, indicating the significance of the mediating effect. Based on these results, the indirect effect is found to be significant, and Hypothesis 2 is supported.

**Table 4:** Results of bootstrapped indirect effect tests.

Variables	Coefficient	SE	CI lower limit	CI upper limit
*Indirect effect (H2)*				
Psychological capital	0.20	0.03	0.14	0.25
*Conditional indirect effect of performance pressure (H3)*				
Low	0.19	0.03	0.13	0.26
High	0.14	0.03	0.89	0.21

Results of the bootstrap iterated 10,000 times are presented; SE, standard error; BC, bias-corrected percentile method; CI, 95% confidence interval.

Third, performance pressure’s moderating effect on the relationship between authentic leadership and psychological capital is explained in this section. Hypothesis 3 argues that performance pressure, as perceived by members, negatively moderates authentic leadership’s influence on psychological capital. To test this, the result of introducing the independent variable and moderator along with the generated interaction term is described in Model 6. The variables were grand mean centered according to the suggestion of Aiken et al. (1991) before generating the interaction term. This was done to prevent multicollinearity issues and to facilitate the analysis. Model 6 reveals that the interaction term’s regression coefficient is significant in the negative direction (*β* = −0.08, *p* < 0.05). Based on this result, Hypothesis 3 is supported.

**Figure 2:**
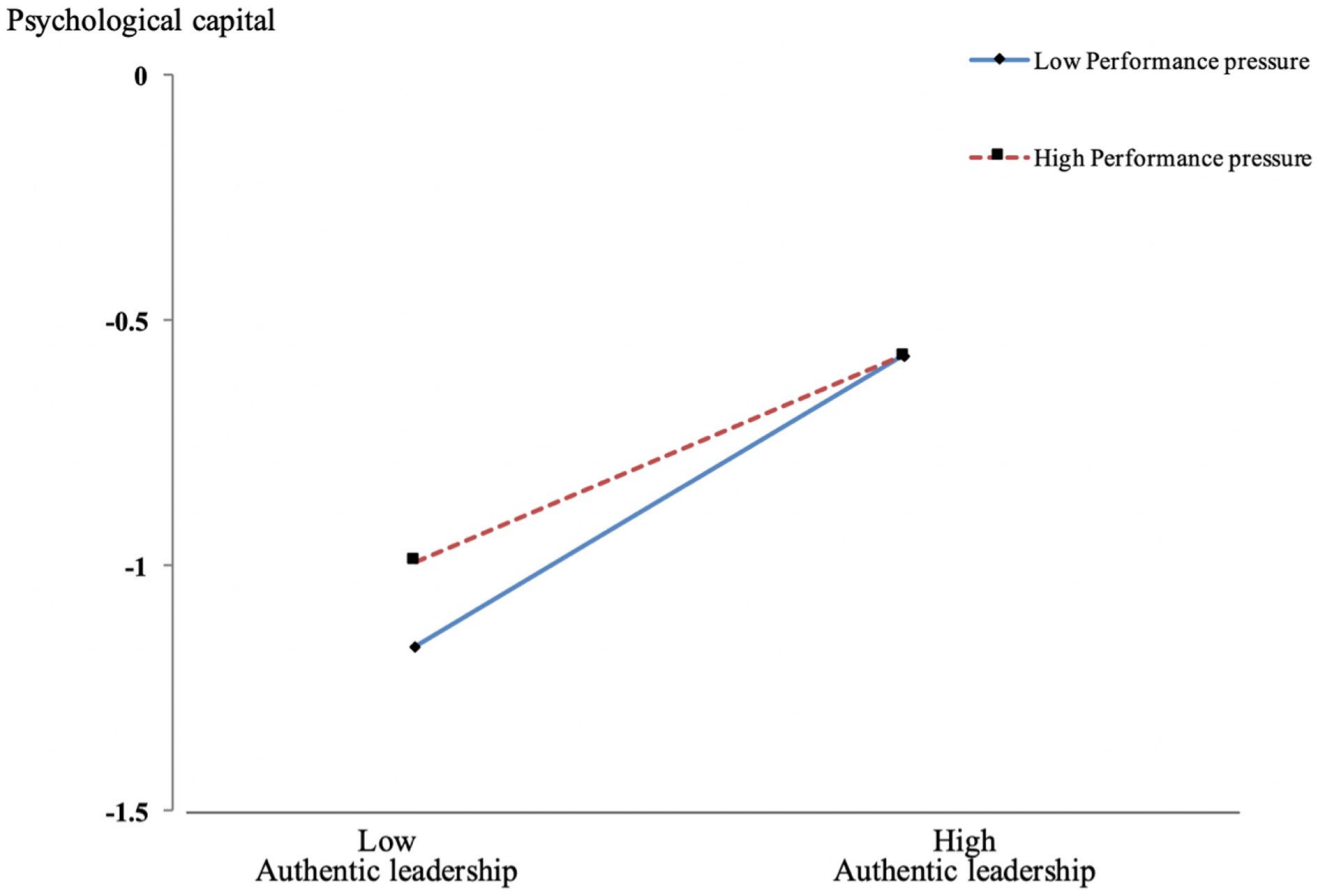
Moderating effect of performance pressure of authentic leadership on the relationship between psychological capital.

Additional *t-*tests were conducted using the method suggested by Aiken et al. (1991) to closely examine the interaction effect of authentic leadership and performance pressure. The significance of the correlation coefficients (simple slope; Aiken et al., 1991) was examined after calculating the large and small one standard deviation values based on performance pressure’s mean and estimating the respective regression equations. The regression coefficients and significance levels vary when the performance pressure’s value is high (*b* = 0.01, n. s.) or low (*b* = 2.67, *p* < 0.05). This demonstrates that a higher level of performance pressure perceived by members leads to authentic leadership’s weaker positive influence on psychological capital, thereby supporting Hypothesis 3 (Table 3, See Figure 2).

To test Hypothesis 4, the bootstrapping approach was used in this study to verify the conditional indirect effect. Table 4 provides the results from the bootstrap approach that was iterated 10,000 times each for the point estimate, standard error, and 95% confidence intervals (percentile and bias-corrected percentile). Under low performance pressure, the point estimate of authentic leadership’s indirect effect on task performance through psychological capital is 0.19 (bootstrap standard error = 0.03), and 0 is not included in the statistically significant bias-corrected 95% confidence interval. Furthermore, under high performance pressure, the point estimate of the indirect effect of authentic leadership on task performance through psychological capital is 0.14 (bootstrap standard error = 0.03), and 0 is not included in the statistically significant bias-corrected 95% confidence interval. The indirect effect’s point estimate decreased from 0.19 to 0.14 when the level of performance pressure changed from “low” to “high.” This result demonstrates that the conditional indirect effect (i.e., the level of performance pressure) weakens authentic leadership’s positive influence on task performance through psychological capital. Moreover, the indirect effect becomes weaker with the interaction’s increasing frequency. Therefore, Hypothesis 4 is supported (i.e., the performance pressure level negatively weakens the strength of the mediated relationship between authentic leadership and task performance through members’ psychological capital).

## Discussion

### Theoretical Implications

This study attempted to verify the influence of authentic leadership as perceived by private domestic enterprises’ members on their task performance as well as the mediating effect of psychological capital and the moderating effect of performance pressure on the relationship between authentic leadership and task performance.

First, authentic leadership positively influences members’ task performance. Many prior studies have found that authentic leadership affects members’ performance (Laschinger et al., 2012; Wang et al., 2014; Leroy et al., 2015), collective performance (Xiong and Fang, 2014), and prosocial behavior (Hannah et al., 2005). However, studies verifying their influence on performance are still limited. Thus, the present study’s first result is meaningful as the argument of authentic leadership’s significant influence on members’ performance (Gardner et al., 2005) is reaffirmed by the members of private enterprises in South Korea. This result can help resolve the myth that authentic leaders are indecisive and friendly (Yoon, 2012).

Second, the mediating effect of psychological capital on authentic leadership’s influence on task performance was demonstrated. The need to study psychological capital as a mediator has been continuously increasing (Avolio et al., 2004; Ilies et al., 2005; Yammarino et al., 2008). In this regard, the demonstration of psychological capital mediating effect on the relationship between authentic leadership and task performance has theoretical significance. Authentic leadership, which is based on authenticity, develops psychological capital consisting of self-efficacy, hope, optimism, and resilience. It influences task performance through intrinsic motivation that improves organizational performance. Psychological capital is much more stable than emotions, which may quickly change. It is also easier to develop than personality and can be maintained. Therefore, psychological capital can contribute to organizational effectiveness from a long-term perspective, even in rapidly changing organizational conditions. In this study, psychological capital’s mediating effect reaffirmed that authentic leadership can contribute to organizational performance by developing the members’ psychological capital and motivating them.

Third, performance pressure moderated authentic leadership, such that the effectiveness of the development of members’ psychological capital was weakened. That is, authentic leadership’s influence on psychological capital development was weaker among members who perceived a high level of performance pressure compared to those who perceived a low level of performance pressure. The present study’s results confirmed that the positive motivation to imitate and follow authentic leaders in the process of developing psychological capital could be hindered by extrinsic control when members perceive a high level of performance pressure. Many researchers have argued that the effectiveness in various organizational situations should be verified by future research on authentic leadership (Gardner et al., 2011; Petersen and Youssef-Morgan, 2018). Performance pressure’s moderating effect as perceived by members was demonstrated in this study. The present study is in line with a prior study (Petersen and Youssef-Morgan, 2018) that argued that authentic leadership’s development to generate sustainable performance could be hampered in an organizational climate governed by performance pressure. The demonstration of performance pressure’s moderating effect is of great theoretical significance as it contributes to a broader and more sophisticated authentic leadership theory.

### Practical Implications

Based on prior research, this study demonstrated the influence of direct supervisors’ authentic leadership, as perceived by various organizations’ members, on task performance and affirmed psychological capital’s mediating effect and performance pressure’s moderating effect in the aforementioned process. The study derived the following practical implications.

First, authentic leadership’s importance should be recognized as it was found to have a significant and positive influence on task performance. Moreover, a higher level of authentic leadership competency should be promoted at the organizational level (Luthans and Avolio, 2003; Avolio and Gardner, 2005; Gardner et al., 2005; Wang et al., 2014). Many previous studies have suggested methods for developing authentic leadership (Avolio, 2010). First, from the content-related aspect of leadership training programs in an organization, participants should be encouraged to sufficiently reflect upon and constantly reviewed concepts, such as authenticity, integrity, and effectiveness (Gardner et al., 2011). To accomplish this, the training program should be redesigned from a methodological perspective, such that authenticity can be developed. As implemented by many organizations, a simple training leadership program alone poses challenges in developing authentic leadership. Therefore, programs specializing in leaders’ characteristics that can encourage authenticity should be developed rather than developing lecture-based programs. For example, a learning opportunity where participants are given time to ponder over life stories and understand the meaning of a series of events (Shamir and Eilam, 2005) can help establish authenticity in leaders. Moreover, such a learning opportunity can improve their capacity as authentic leaders. Furthermore, it is a great learning opportunity to discuss how authenticity attracts voluntary followership by reflecting on a third leader who is perceived to exhibit authenticity by a participating leader.

Second, psychological capital’s mediating effect demonstrates that authentic leadership is closely related to psychological capital. An important characteristic of psychological capital is that it can also be developed through an intervention like short-term training. Therefore, it is reiterated that sustainable performance can be achieved in a rapidly changing business environment by developing authentic leadership in an organization and advancing members’ psychological capital.

Third, when an organization seeks to improve performance through the development of authentic leadership, the perceived level of performance pressure within the organization needs to be considered. Moreover, focusing solely on the performance of the enterprise should be avoided. The perception of excessive performance pressure serves as a motivation for members to momentarily avoid external threats, hindering the formation of a healthy organization and exerting negative impact on leaders. Pressuring leaders to succeed in a short period and prove themselves impairs balanced information processing in the development of authentic leadership (Petersen and Youssef-Morgan, 2018). Extra caution should be exercised in an organization that focuses too much on short-term performance if long-term and sustainable performance is desired.

Alternative solutions are also required to ensure that performance pressure does not reduce authentic leadership’s effectiveness. In actual business management, it may be unrealistic to avoid performance pressure because an organization must survive by maximizing productivity (Mitchell et al., 2018). To compensate for this, it is necessary to emphasize on not only quantitative performance, but also ethical practices as part of the performance structure. Such efforts of an organization to manage performance will lay the foundation for fostering more authentic leadership. Leadership that lacks authenticity can still have a positive influence on performance (Fu et al., 2010; Yoon, 2012). However, it can create organizational inequality and corruption in the long run. Therefore, performance pressure should be managed at an organizational level, such that authentic leadership’s positive influence is not compromised but rather multiplied. Ultimately, competitive advantages at the individual, team, and organizational levels depend on developing a high level of leadership. It should be noted that authentic leadership can produce sustainable performance in today’s challenging business environment.

### Limitations and Future Research

The hypotheses proposed in this study were accepted based on the empirical analysis results. Moreover, meaningful implications from a practical perspective were also derived. Nevertheless, suggestions were made to address the following limitations.

First, while performance pressure was viewed in a negative light, both negative and positive characteristics were noted in prior studies (Gardner, 2012; Mitchell et al., 2018). Therefore, future research should consider both aspects. Regarding performance pressure’s moderating effect, when authentic leadership’s influence was small, members who perceived a high level of performance pressure had greater psychological capital compared to those who perceived a low level of performance pressure. Moreover, there may be other moderators in addition to authentic leadership that can affect psychological capital. Apart from authentic leadership, Avey (2014) stated that personal characteristics and job structure are antecedents that could influence psychological capital. Therefore, a broader understanding of the mechanism of authentic leadership can be achieved if future research is designed and verified by considering both performance pressure and psychological capital.

Second, it is necessary to prove the presence of transformational leadership’s additional influence despite controlling it during the verification of authentic leadership’s influence. An important feedback in authentic leadership’s theoretical development stems from transformational leadership’s retrospective criticism (Michie and Gooty, 2005). Strong correlations between authentic leadership and transformational leadership have been reported through a meta-analysis of the two concepts (Banks et al., 2016). Therefore, it is possible to demonstrate the extent of authentic leadership’s isolated effect by verifying authentic leadership’s effectiveness in controlling transformational leadership.

Finally, the self-report method by the same respondents raised concerns about CMB. This is inevitable to some degree in leadership research as surveys based on members’ perceptions are effective (Oc and Bashshur, 2013). Moreover, it is only valid for gauging the level of psychological capital that members themselves perceive to possess (Walumbwa et al., 2010). However, authentic leadership’s influence on psychological capital and performance can be confirmed in future research by using a method wherein members evaluate their leaders’ authentic leadership, while the leaders evaluate performance as a dependent variable. Furthermore, the causal relationship between authentic leadership, psychological capital, and performance needs to be clarified by designing scenario-based experiments.

We hope that these suggestions will be considered to resolve many of the limitations raised in this study. We also look forward to encouraging more researchers to investigate the relationship between authentic leadership and performance and to actively undertake research that will contribute both academically and practically in the future.

## Conclusion

The study argues that the construct of authentic leadership, which originated in 2004 and has been studied as an essential leadership theory for about the last 20 years, needs to consider situational factors in order to be established as a more sophisticated leadership theory (Gardner et al., 2011, 2021; Petersen and Youssef-Morgan, 2018). The study results reconfirmed that authentic leadership positively affects psychological capital, thereby enhancing employee task performance. This supports the argument that subordinates respond to leader authenticity by generating better performance as an individual (e.g., Wang et al., 2014; Ribeiro et al., 2018a). Furthermore, a new variable, namely, short-term performance pressure, was demonstrated as an organizational climate variable that weakens the effect of authentic leadership on psychological capital. This implies that sustainable authentic leadership from a long-term perspective can hinder development in an atmosphere that focuses on short-term results. This study contributes to the deepening of the literature on authentic leadership, psychological capital, and task performance by examining the situational factor of short-term performance pressure, which has hardly been investigated previously.

## Data Availability Statement

The original contributions presented in the study are included in the article/supplementary material, further inquiries can be directed to the corresponding author.

## Ethics Statement

Ethical review and approval was not required for the study on human participants in accordance with the local legislation and institutional requirements. The patients/participants provided their written informed consent to participate in this study.

## Author Contributions

The author confirms sole responsibility for the following: study conception and design, data collection, analysis and interpretation of results, and manuscript preparation.

## Conflict of Interest

The author declares that the research was conducted in the absence of any commercial or financial relationships that could be construed as a potential conflict of interest.

## Publisher’s Note

All claims expressed in this article are solely those of the authors and do not necessarily represent those of their affiliated organizations, or those of the publisher, the editors and the reviewers. Any product that may be evaluated in this article, or claim that may be made by its manufacturer, is not guaranteed or endorsed by the publisher.
